# Associations Between Cannabis Use, Methylphenidate Exposure, and Radiographically Detected Pulp Stones: An Exploratory Case‐Control Study

**DOI:** 10.1155/ijod/5655140

**Published:** 2026-08-24

**Authors:** Michael Saminsky, Aliya Ben-David, Evgenia Balaban, Iris Manor, Tomer Goldberger

**Affiliations:** ^1^ Department of Periodontology and Oral Implantology, Goldschleger School of Dental Medicine, Gray Faculty of Medical and Health Sciences, Tel Aviv University, Tel Aviv, Israel, tau.ac.il; ^2^ Dan-Petach-Tikva District, Clalit Health Services, Petach Tikva, Israel, clalit.co.il; ^3^ Gray Faculty of Medicine and Health Sciences, Tel Aviv University, Tel Aviv, Israel, tau.ac.il; ^4^ Geha Mental Health Center, Petach Tikva, Israel, geha.com; ^5^ Department of Endodontology, Goldschleger School of Dental Medicine, Gray Faculty of Medical and Health Sciences, Tel Aviv University, Tel Aviv, Israel, tau.ac.il

**Keywords:** ADHD, bruxism, cannabis, dental pulp stones, methylphenidate, parafunctional habits

## Abstract

**Aims:**

This exploratory study investigated the associations between cannabis use, methylphenidate (MPH) exposure, demographic factors, and radiographically detected pulp stones.

**Materials and Methods:**

A retrospective case‐control study was conducted to analyze the records of 200 adults (100 with pulp stones and 100 controls) treated at a university dental clinic (2020–2023). The variables included age, sex, cannabis use, MPH use, tobacco smoking, and clenching/grinding behaviors. Analyses included Fisher’s exact test, Mann–Whitney *U* test, and multivariable logistic regression with Benjamini–Hochberg (BH) correction.

**Results:**

Cannabis use showed the strongest independent association with radiographic pulp stones (OR = 6.54, 95% CI: 1.95–21.95; BH‐corrected *p* = 0.012). Age was also independently associated (OR = 1.08/year; BH‐corrected *p* = 0.035). MPH use was associated with pulp stones on unadjusted analysis (OR = 2.3, *p* = 0.042), but not after adjustment (OR = 1.72; BH‐corrected *p* = 0.232). MPH users demonstrated significantly higher rates of documented clenching/grinding behaviors than nonusers (41.7% vs. 23.8%; *p* = 0.038). Tobacco smoking was not associated with the presence of pulp stones.

**Conclusions:**

Cannabis use was the strongest independent factor associated with radiographically detected pulp stones. MPH users showed higher rates of clenching/grinding behaviors, supporting a possible mechanistic relationship between psychostimulant exposure and parafunctional activity. The findings should be considered exploratory and hypothesis‐generating.

**Clinical Significance:**

Pulp stones may be radiographic indicators of underlying parafunctional activity and behavioral risk factors, supporting more comprehensive dental history‐taking and monitoring strategies.

**Summary**

Cannabis use demonstrated the strongest independent association with radiographically detected pulp stones, methylphenidate users showed significantly higher rates of parafunctional clenching/grinding behaviors, and pulp stones in intact teeth may represent a radiographic marker warranting assessment of behavioral and medication‐related risk factors.

## 1. Introduction

Pulp stones (denticles) are calcified formations within the dental pulp that can complicate endodontic treatment by reducing pulp cell populations and impeding canal instrumentation [1]. Radiographic studies estimate their prevalence at ~15% of teeth, although histological studies report substantially higher rates [1, 2]. Their etiology is multifactorial and may involve local factors, including aging, trauma, and chronic irritation, as well as systemic conditions, such as cardiovascular disease, diabetes, and kidney stones [3–9]. Excessive parafunctional occlusal forces have also been proposed as a contributing mechanism [10–12]. Despite these proposed mechanisms, the influence of modifiable behavioral and pharmacological exposures on pulp stone formation remains poorly understood.

Cannabis use has been associated with an increased risk of parafunctional clenching/grinding behaviors [13], providing a biologically plausible pathway linking cannabis exposure to pulpal calcification. Cannabinoid receptors have also been identified in the dental pulp tissue, suggesting a potential direct interaction between cannabinoid signaling and pulpal physiology [14]. Additionally, the available evidence regarding the association between tobacco smoking and parafunctional clenching/grinding behaviors remains inconsistent [15, 16].

Methylphenidate (MPH), a dopamine and noradrenaline reuptake inhibitor, is among the most widely prescribed medications for attention‐deficit/hyperactivity disorder (ADHD), a neurodevelopmental disorder affecting ~7.2% of children and adolescents and 2.5% of adults [17–21]. MPH use has also been associated with bruxism and secondary clenching/grinding behaviors, although the causal evidence remains limited [22–27]. Cannabis use is common among individuals with ADHD who are prescribed MPH [28], suggesting that these exposures frequently coexist. However, their potential relationship with pulp stone formation has not been investigated. Clarifying these associations could improve our understanding of potentially modifiable risk factors relevant to the dental practice.

This exploratory case‐control study aimed to investigate the association between cannabis use and radiographically detected pulp stones. Secondary objectives were to examine the associations of MPH use, parafunctional clenching/grinding behaviors, and demographic factors with the pulp stone presence.

## 2. Materials and Methods

This retrospective case‐control study was based on clinical and radiographic records from the Tel Aviv University School of Dental Medicine (2020–2023). Ethical approval was obtained (Confirmation Number 1‐0007743). The study was conducted in accordance with the STROBE guidelines.

### 2.1. Inclusion and Exclusion Criteria

Inclusion criteria: Available periapical and bitewing radiographs demonstrating the premolars and molars included in the study; age ≥18 years; intact premolars and molars (i.e., no caries, restorations, crowns, or endodontic treatment in the evaluated teeth); and functional antagonist teeth.

Exclusion criteria: Psychiatric medications with known clenching/grinding behavior side effects (SSRIs, antipsychotics, and tricyclic antidepressants); history of orthodontic treatment; and bruxism‐related behaviors with documented onset prior to MPH initiation.

Medical history was obtained from a standardized patient questionnaire and verified using an up‐to‐date medical summary provided by the patient’s family physician, which was reviewed and archived in the institutional dental record. Systemic conditions of interest included cardiovascular disease, diabetes mellitus, chronic kidney disease, and kidney stones. None of the included subjects had any documented evidence of these conditions.

### 2.2. Data Collection

Demographic, medication, and lifestyle data were extracted from the medical records. Participants were contacted where necessary to clarify the MPH dose, regimen, and onset of clenching/grinding behaviors relative to medication initiation. Bruxism was identified using the standardized tool for the assessment of bruxism (STAB) [29], which was documented and additionally via direct patient contact. One hundred records with radiographic pulp stones were designated as the study group, and 100 records without pulp stones formed the control group.

### 2.3. Radiographic Analysis

**Figure 1:**
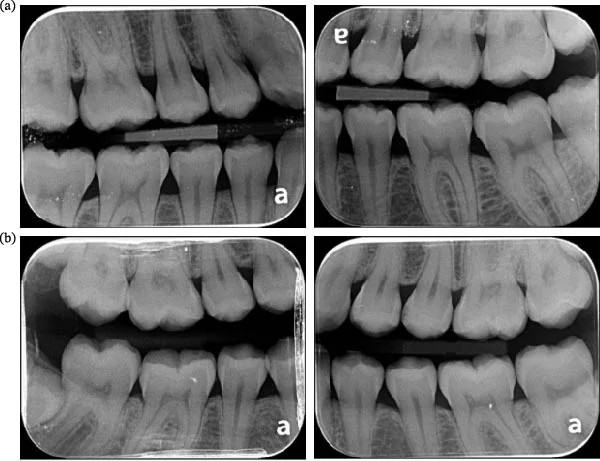
Radiographic appearance of pulp stones. Representative bitewing radiographs from study participants demonstrating radiopaque pulp calcifications (pulp stones) within the pulp chambers. (a) Bilateral first and second maxillary molars and bilateral first mandibular molars exhibiting pulp stones in a participant with documented cannabis use and clenching/grinding behaviors. (b) Bilateral first and second maxillary molars, the right shows first and second mandibular molars, and the left shows first mandibular molar exhibiting pulp stones in a participant with documented cannabis use, methylphenidate (MPH) exposure, and clenching/grinding behaviors.

Radiographic assessment for pulp stones was limited to premolars and molars, which constituted the teeth included in the study analysis. All available periapical and bitewing radiographs demonstrating these teeth were reviewed. All eligible premolars and molars were examined in each participant. The participant, rather than the individual tooth, was considered the unit of analysis. Participants were classified as having radiographic pulp stones only when bilateral pulp stones were identified in at least four of the evaluated teeth. Representative radiographs demonstrating radiographic pulp stones are shown in Figure 1a,b. The radiographs were obtained retrospectively during routine patient care using a Planmeca ProX intraoral X‐ray unit (Planmeca Oy, Helsinki, Finland). Images were acquired using ScanX Duo photostimulable phosphor (PSP) imaging plates and a scanner (Air Techniques, Melville, NY, USA) and processed using MediaDent software. Exposure parameters were standardized at 70 kVp, 8 mA, and 0.160 s. Image assessment was performed by a single examiner (T.G., a specialist in endodontics) using MediaDent software on a 22‐inch NEC MultiSync E224Wi flat‐screen monitor (1920 × 1080‐pixel resolution) at ×2 magnification in a designated darkened room with no ambient illumination other than the monitor. Brightness and contrast were not adjusted, and no digital edge‐enhancement filters or other image‐processing tools were applied during image evaluation. To assess intraexaminer consistency, 10 randomly selected radiographs were reevaluated at a separate time point, yielding identical findings in all repeated assessments. Formal reliability statistics were not calculated because complete concordance was observed within the limited calibration subset. No independent second examiner was involved in the radiographic assessment.

### 2.4. Statistical Analysis

Data were analyzed using SPSS v29.0 and R v4.4.0. Univariate associations between categorical variables and group membership were assessed using Fisher’s exact test; continuous variables were compared with the Mann–Whitney *U* test. Multivariable analysis used binomial logistic regression. Age, sex, tobacco smoking, and MPH use were treated as potential confounders and included in the multivariable regression model. No a priori effect modifiers were specified, and interaction analyses were not performed. Multiple comparisons were corrected using the Benjamini–Hochberg (BH) procedure.

Exploratory analyses included comparison of the prevalence of documented clenching/grinding behaviors between MPH users and nonusers using Fisher’s exact test and a sensitivity analysis evaluating the association between cannabis use and radiographically detected pulp stones after excluding participants receiving MPH therapy. Given the modest number of MPH and cannabis users, the regression analyses were considered exploratory and intended primarily for effect size estimation and hypothesis generation rather than definitive causal inference.

## 3. Results

### 3.1. Sample Characteristics

Two hundred subjects were included (100 per group). The study group comprised 58 females and 42 males, a mean age of 28.9 years (SD = 4.9), and the control group comprised 46 females and 54 males, a mean age of 27.3 years (SD = 4.8). Groups did not differ significantly in sex distribution (*p* = 0.120, Fisher’s exact test) but did differ in age (*p* = 0.017, Mann–Whitney *U* test). Tobacco smoking was reported in 25 and 23 subjects in the study and control groups, respectively (*p* = 0.869). None of the subjects had cardiovascular disease, diabetes mellitus, chronic kidney disease, or kidney stones. Sample characteristics are summarized in Table 1.

**Table 1:** Demographic and lifestyle characteristics of the study and control groups.

Variable	Study group pulp stones (*n* = 100)	Control group no pulp stones (*n* = 100)	*p*‐Value
Sex (female/male)	58/42	46/54	0.120 ^∗^
Mean age, years (SD)	28.9 (4.9)	27.3 (4.8)	0.017^†^
Tobacco smoking, *n* (%)	25 (25%)	23 (23%)	0.869 ^∗^
MPH use, *n* (%)	24 (24%)	12 (12%)	0.042 ^∗^
Immediate release, *n*	8	3	0.213 ^∗^
Slow release, *n*	17	10	0.214 ^∗^
Cannabis use, *n* (%)	19 (19%)	4 (4%)	0.001 ^∗^
Bruxism/clenching, *n* (%)	30 (30%)	24 (24%)	0.426 ^∗^

*Note:* Immediate‐release and slow‐release methylphenidate categories are not mutually exclusive. Two participants (one case and one control) received both formulations during the study period; therefore, the sum of the formulation categories exceeds the total number of methylphenidate users. Age *p*‐value reflects a significant difference between groups and is included as a covariate in all regression models.

Abbreviation: MPH, methylphenidate.

^∗^Fisher’s exact test.

^†^Mann–Whitney *U* test.

### 3.2. Unadjusted Associations

On univariate analysis, MPH use was significantly more prevalent in the study group (24%) than controls (12%; Fisher’s *p* = 0.042). Neither MPH release mode (IR: *p* = 0.213; SR: *p* = 0.214), dose category (high dose: *p* = 0.537; low dose: *p* = 0.097), nor MPH duration (cases: median 9 months, IQR 1–39; controls: median 12 months, IQR 8–27; *p* = 0.531) differed significantly between groups. Cannabis use was strongly associated with pulp stone presence: 19 cases versus 4 controls (Fisher’s *p* = 0.001). Further review of the cannabis‐user subgroup revealed that all participants reporting cannabinoid use described cannabis consumption by smoking and reported use for at least 3 months. However, detailed information regarding frequency of use, cumulative exposure, dose, and cannabis potency was not consistently available and could not be reliably quantified. Bruxism was present in 30 cases and 24 controls (*p* = 0.426).

### 3.3. Multivariable Logistic Regression

In the multivariable model (Table 2), cannabis use was the only highly significant independently associated factor after BH correction (OR = 6.54, 95% CI: 1.95–21.95; BH‐corrected *p* = 0.012). Age was independently associated (OR = 1.08 per year, 95% CI: 1.02–1.15; BH‐corrected *p* = 0.035). Female sex showed a borderline trend (OR = 1.94, 95% CI: 1.05–3.58; raw *p* = 0.033; BH‐corrected *p* = 0.056). MPH use did not reach significance in the adjusted model (OR = 1.72, 95% CI: 0.77–3.86; BH‐corrected *p* = 0.232). Tobacco smoking was not associated with pulp stone presence (OR = 0.74; BH‐corrected *p* = 0.436).

**Table 2:** Multivariable binomial logistic regression: likelihood of radiographic pulp stone presence.

Variable	Estimate	Std. error	OR (95% CI)	*p* (raw)	*p* (BH)
Female sex	0.663	0.312	1.94 (1.05–3.58)	0.033	0.056
Age (per year)	0.080	0.032	1.08 (1.02–1.15)	0.014	0.035 ^∗^
Cannabis use	1.878	0.618	6.54 (1.95–21.95)	0.002	0.012 ^∗^
MPH use	0.545	0.412	1.72 (0.77–3.86)	0.186	0.232
Tobacco smoking	0.545	0.383	0.74 (0.35–1.57)	0.436	0.436

*Note:* Reference categories: male sex; no tobacco use; no cannabis use; and no MPH use.

Abbreviations: BH, Benjamini–Hochberg correction; CI, confidence interval; OR, odds ratio.

^∗^Statistically significant after BH correction (*p* < 0.05).

MPH users demonstrated significantly higher rates of documented clenching/grinding behaviors than nonusers (41.7% vs. 23.8%; Fisher’s exact test, *p* = 0.038). To evaluate whether the association between cannabis use and radiographically detected pulp stones was independent of MPH exposure, a sensitivity analysis restricted to participants not using MPH (76 cases and 88 controls) was performed. Cannabis use remained significantly associated with pulp stone presence (12 cases vs. 3 controls; OR = 5.31, *p* = 0.007), consistent with the primary multivariable analysis.

## 4. Discussion

The principal finding of this exploratory study is that cannabis use demonstrated the strongest independent association with radiographic pulp stone prevalence, with an ~6.5‐fold increased odds relative to nonusers after adjustment for age, sex, tobacco use, and MPH use. This association is biologically plausible as cannabis has been shown to increase the risk of parafunctional clenching/grinding behaviors [13], and excessive parafunctional occlusal forces are a recognized contributor to pulpal calcification [10–12]. To the best of our knowledge, this is among the first studies to report such an association between cannabis use and dental pulp stones.

MPH use was associated with pulp stones on unadjusted analysis (OR = 2.3, *p* = 0.042), consistent with the hypothesis that stimulant‐induced parafunctional activity may contribute to pulpal calcification. However, this association did not survive multivariable adjustment (OR = 1.72; BH‐corrected *p* = 0.232). Notably, the adjusted cannabis OR (6.54) exceeded the crude OR (5.63), indicating that adjustment for MPH did not inflate the cannabis estimate—if anything, MPH acted as a mild negative confounder rather than a source of spurious amplification. As a further sensitivity check, restricting the analysis to subjects not using MPH (76 cases, 88 controls) yielded a cannabis OR of 5.31 (12 cases vs. 3 controls; *p* = 0.007), corroborating the robustness of the primary finding independently of MPH coexposure. The unadjusted association between MPH and pulp stones should therefore be interpreted cautiously as a preliminary signal warranting investigation in a larger, adequately powered study.

### 4.1. Cannabis and Pulp Stone Formation

The association between cannabis use and pulp stones warrants specific attention. Several mechanisms may be operating. Cannabis has been associated with increased parafunctional clenching/grinding behaviors [13], and the resulting excessive occlusal forces may stimulate pulpal calcification. Cannabinoid receptors have additionally been identified in dental pulp tissues, suggesting a potential biological interaction between cannabinoid signaling and pulpal physiology [14]. Supporting this, in vitro evidence shows that cannabidiol enhances viability, proliferation, and odontogenic differentiation of human dental pulp stem cells, upregulating DSPP, RUNX2, and osteocalcin via MAPK and WNT/β‐catenin pathways, indicating regenerative potential in endodontic contexts [30]. At the mechanistic level, odontoblast studies demonstrate functional coupling between CB1 receptors and TRPV1‐mediated Ca^2+^ signaling, integrated with Na^+^/Ca^2+^ exchangers that regulate intracellular calcium dynamics during stimulus response, suggesting a role for cannabinoid‐TRPV1‐NCX interactions in dentinogenic signaling [31].

Although the clinical implications of these findings remain uncertain, they provide biological plausibility for the observed association. The independence of the cannabis association from MPH coexposure is supported by two observations: first, the adjusted cannabis OR (6.54) exceeded the crude OR (5.63), demonstrating that adjustment for MPH did not spuriously inflate the cannabis estimate; and second, in a sensitivity analysis restricted to subjects with no MPH use (76 cases, 88 controls), cannabis remained significantly associated with pulp stones (12 cases vs. 3 controls; OR = 5.31; *p* = 0.007). Together, these findings support a direct association between cannabis use and pulp stone presence that is not attributable to MPH coexposure.

### 4.2. MPH Use and Parafunctional Activity

MPH users demonstrated significantly higher rates of documented clenching/grinding behaviors compared with nonusers (41.7% vs. 23.8%; Fisher’s *p* = 0.038). This finding is consistent with established pharmacological mechanisms: as a dopaminergic and noradrenergic reuptake inhibitor, MPH may increase jaw muscle activity through central dopaminergic stimulation [25, 26]. One study reported a 1.67‐fold increased risk of sleep bruxism in adolescents using MPH [23], and the present findings extend this signal to the adult population.

However, the interpretation of this mechanistic pathway requires caution. Assessment of clenching/grinding behaviors relied primarily on clinical documentation, STAB records, where available, and patient self‐report supplemented by direct patient contact. Such retrospective ascertainment is susceptible to recall bias and incomplete documentation, particularly regarding the temporal relationship between medication exposure and parafunctional behaviors. Objective measures such as polysomnography were not available. Consequently, the observed association between MPH use and parafunctional activity should be regarded as supportive but not definitive evidence of a biological pathway linking psychostimulant exposure and pulpal calcification.

### 4.3. Demographic Predictors

Age was independently associated with pulp stone presence (OR = 1.08 per year), consistent with the established increase in pulp stone prevalence with age attributed to cumulative pulpal stimuli and progressive pulpal degeneration [32]. Female sex showed a borderline trend after BH correction (BH‐corrected *p* = 0.056). This finding is consistent with reports of higher rates of bruxism, anxiety, and stress among women [33], as well as recent metaanalytic evidence identifying female sex as a predictor of bruxism prevalence [34].

Tobacco smoking was not associated with pulp stone presence in this cohort, consistent with evidence suggesting that parafunctional activity is not consistently related to the smoking status [16].

### 4.4. Strengths and Limitations

This study is, to the best of our knowledge, among the first to investigate the association between cannabis use and radiographic pulp stone prevalence. Additional strengths include strict inclusion and exclusion criteria that minimized local pulpal confounders by restricting the radiographic assessment to intact premolars and molars; standardized image acquisition and evaluation using radiographs under controlled viewing conditions; and multivariable regression modeling with adjustment for relevant confounders and correction for multiple comparisons. The consistency of the primary association across adjusted and sensitivity analyses further supports the robustness of the findings.

Several limitations should nonetheless be acknowledged. First, the retrospective cross‐sectional design precludes causal inference or the establishment of temporal precedence, and the samples of MPH users (*n* = 36) and cannabis users (*n* = 23) were relatively small, increasing uncertainty around regression estimates. However, the 95% confidence interval for the primary cannabis association (1.95–21.95) excluded the null value, and the association remained statistically significant both before (*p* = 0.002) and after BH correction (*p* = 0.012).

Second, exposure characterization had some constraints. Cannabis use was recorded as a binary variable, and detailed data on frequency, cumulative exposure, dose, and potency were not consistently available, precluding dose‐response analysis. Cannabis and MPH exposure also overlapped in 8 of the 23 cannabis users; this was addressed by a sensitivity analysis restricted to non‐MPH users. Additionally, cannabis use and parafunctional clenching/grinding behaviors were ascertained from retrospective clinical records, supplemented where necessary by direct patient contact, introducing the potential for recall bias, underreporting, or exposure misclassification. Assessment of parafunctional clenching/grinding behaviors relied on clinical documentation, STAB records where available, and patient self‐report rather than objective methods such as polysomnography. Consequently, bruxism was not included as a covariate in the multivariable regression model, and the primary association between cannabis use and pulp stone presence was derived independently of the bruxism assessment.

Third, residual confounding cannot be fully excluded. Several additional local and systemic factors, including periodontal disease, previous dental trauma, dental bleaching procedures, and statin therapy, have been proposed as potential contributors to pulp stone formation. Because these variables were not systematically recorded in the retrospective clinical records, they could not be incorporated into the eligibility criteria or adjusted for in the statistical analyses. Consequently, residual confounding cannot be excluded, and the observed associations should be interpreted as exploratory. Future prospective studies should systematically collect these variables to better define their independent contribution to pulp stone formation.

Fourth, radiographic outcome assessment also has inherent limitations. Pulp stone identification was based exclusively on radiographs, which may underestimate the true prevalence relative to histological examination because small calcifications, anatomical superimposition, and image‐quality limitations may prevent the detection of some pulp stones. Consequently, the prevalence estimates reported in this study should be interpreted as radiographic rather than histological prevalence. Radiographic assessment was also performed by a single examiner without independent interexaminer validation. Although repeated evaluation of a small calibration subset yielded identical findings, formal reliability statistics (e.g., kappa coefficients) were not calculated; therefore, some degree of observer bias or misclassification cannot be excluded.

Future prospective studies involving larger, well‐characterized cohorts should incorporate standardized assessment of cannabis exposure, objective evaluation of parafunctional clenching/grinding behaviors (e.g., polysomnography), comprehensive assessment of additional local and systemic risk factors, and longitudinal radiographic follow‐up before and after medication exposure to better clarify the temporal and biological relationships underlying pulp stone formation.

## 5. Conclusions

Within the limitations of this exploratory retrospective case‐control study, cannabis use demonstrated the strongest independent association with radiographically detected pulp stones (OR = 6.54; BH‐corrected *p* = 0.012), with age acting as an additional independent contributor. MPH use showed a biologically plausible unadjusted association that did not survive multivariable adjustment, likely reflecting limited statistical power and collinearity between exposures. MPH users additionally demonstrated higher rates of documented clenching/grinding behaviors, supporting a potential mechanistic relationship between psychostimulant exposure and parafunctional activity. These findings should be considered exploratory and hypothesis‐generating and require confirmation in larger prospective studies with standardized behavioral assessment.

### 5.1. Clinical Relevance

This study provides preliminary evidence that cannabis use may represent a clinically relevant risk factor for radiographic pulp stone formation—an association not previously reported. Dental practitioners should consider inquiring about cannabis use when incidental pulp stones are identified, particularly in younger individuals with otherwise intact dentitions. Clinicians prescribing MPH should additionally be aware of its documented association with parafunctional clenching/grinding behaviors, and appropriate dental monitoring or preventive strategies may be considered. Although pulp stones in intact teeth may not have immediate clinical consequences, they may serve as radiographic indicators of underlying parafunctional activity, requiring clinical attention.

## Author Contributions

Conceptualization: Michael Saminsky and Tomer Goldberger. Project administration: Iris Manor, Aliya Ben‐David, and Michael Saminsky. Investigation: Tomer Goldberger, Aliya Ben‐David, and Michael Saminsky. Data curation: Aliya Ben‐David, Evgenia Balaban, and Tomer Goldberger. Validation: Michael Saminsky and Aliya Ben‐David. Formal analysis: Evgenia Balaban and Michael Saminsky. Writing – original draft: Michael Saminsky, Iris Manor, Aliya Ben‐David, and Tomer Goldberger. Writing – review and editing: all authors.

## Funding

This study did not receive any specific funding.

## Disclosure

All authors have read and approved the final version of the manuscript. Michael Saminsky had full access to all the data in this study and takes complete responsibility for the integrity of the data and the accuracy of the data analysis.

## Ethics Statement

Subjects, or their relative/guardians, where applicable, signed an informed consent, which included the information that their radiographic data could be used for future research. Data was anonymized, and the study protocol was reviewed and approved by the Tel Aviv University Ethics Committee (Confirmation Number 1‐0007743).

## Conflicts of Interest

The authors declare no conflicts of interest.

## Data Availability

The data supporting the findings of this study are not publicly available because they comprise sensitive patient radiographic records subject to institutional, ethical, and data protection requirements. Access may be considered upon reasonable request from the corresponding author, subject to institutional approval and applicable privacy regulations.

## References

[1] Kannan S. , Kannepady S. K. , Muthu K. , Jeevan M. B. , and Thapasum A. , Radiographic Assessment of the Prevalence of Pulp Stones in Malaysians, Journal of Endodontics. (2015) 41, no. 3, 333–337, 10.1016/j.joen.2014.10.015. 25476972

[2] Ravichandran S. and Vadivel J. K. , Prevalence of Pulp Stones in IOPA Radiographs, Journal of Advanced Pharmaceutical Technology & Research. (2022) 13, no. Suppl 1, S63–S66, 10.4103/japtr.japtr_126_22. 36643118 PMC9836167

[3] Goga R. , Chandler N. P. , and Oginni A. O. , Pulp Stones: A Review, International Endodontic Journal. (2008) 41, no. 6, 457–468, 10.1111/j.1365-2591.2008.01374.x. 18422587

[4] Nanci A. , Ten Cate’s Oral Histology, 2024, 10th edition, Elsevier.

[5] Memon M. , Kalhoro F. A. , Shams S. , and Arain S. , Pulp Stone: A Study on Radiographic Assessment, The Professional Medical Journal. (2018) 25, no. 7, 992–996, 10.29309/TPMJ/18.3756.

[6] Srivastava K. C. , Shrivastava D. , and Nagarajappa A. K. , et al.Assessing the Prevalence and Association of Pulp Stones With Cardiovascular Diseases and Diabetes Mellitus in Saudi Arabia, International Journal of Environmental Research and Public Health. (2020) 17, no. 24, 10.3390/ijerph17249293, 9293. 33322604 PMC7764339

[7] Alamoudi R. A. , Alzayer F. M. , Alotaibi R. A. , Alghamdi F. , and Zahran S. , Assessment of the Correlation Between Systemic Conditions and Pulp Canal Calcification, Cureus. (2023) 15, no. 9, 10.7759/cureus.45484, 45484. PMC10583123 37859878

[8] Beytaş Alğan B. , Koçak M. M. , Koçak S. , and Sağlam B. C. , Association Between Pulp Stones and Systemic Diseases: A Retrospective Study Using Digital Panoramic Radiographs in a Turkish Population, BMC Oral Health. (2025) 25, no. 1, 10.1186/s12903-025-06492-3, 1134. 40634921 PMC12243179

[9] Bains S. K. , Bhatia A. , Singh H. P. , Biswal S. S. , Kanth S. , and Nalla S. , Prevalence of Coronal Pulp Stones and Its Relation With Systemic Disorders in Northern Indian Central Punjabi Population, ISRN Dentistry. (2014) 2014, 10.1155/2014/617590, 617590. 24944821 PMC4040191

[10] Lobbezoo F. , Van Der Zaag J. , Van Selms M. K. A. , Hamburger H. L. , and Naeije M. , Principles for the Management of Bruxism, Journal of Oral Rehabilitation. (2008) 35, no. 7, 509–523, 10.1111/j.1365-2842.2008.01853.x. 18557917

[11] Landay M. A. and Seltzer S. , The Effects of Excessive Occlusal Force on the Pulp, Oral Surgery, Oral Medicine, Oral Pathology. (1971) 32, no. 4, 623–638, 10.1016/0030-4220(71)90330-6. 5285702

[12] Büyk C. and Arsan B. , Radiomorfometric Analysis of Dental and Trabeculae Bone Changes in Bruxism Patients, European Annals of Dental Sciences. (2023) 50, no. 1, 1–7, 10.52037/eads.2023.0001.

[13] Le A. , Khoo E. , and Palamar J. J. , Associations Between Oral Health and Cannabis Use Among Adolescents and Young Adults, International Journal of Environmental Research and Public Health. (2022) 19, no. 22, 10.3390/ijerph192215261, 15261. 36429978 PMC9691037

[14] Qi X. , Liu C. , and Li G. , et al.Evaluation of Cannabinoids on the Odonto/Osteogenesis in Human Dental Pulp Cells in Vitro, Journal of Endodontics. (2021) 47, no. 3, 444–450, 10.1016/j.joen.2020.12.005. 33352148

[15] Frosztega W. , Wieckiewicz M. , and Nowacki D. , et al.Polysomnographic Assessment of Effects of Tobacco Smoking and Alcohol Consumption on Sleep Bruxism Intensity, Journal of Clinical Medicine. (2022) 11, no. 24, 10.3390/jcm11247453, 7453. 36556069 PMC9785944

[16] Saracutu O. I. , Manfredini D. , and Bracci A. , et al.Awake Bruxism Is Unrelated to Smoking, Despite the Different Psychological Status: A Pilot Study, Oral Diseases. (2025) 31, no. 7, 2323–2330, 10.1111/odi.15270. 39888125 PMC12319381

[17] Chaulagain A. , Lyhmann I. , and Halmøy A. , et al.A Systematic Meta-Review of Systematic Reviews on Attention Deficit Hyperactivity Disorder, European Psychiatry. (2023) 66, no. 1, 10.1192/j.eurpsy.2023.2451, e90. 37974470 PMC10755583

[18] Ougrin D. , Chatterton S. , and Banarsee R. , Attention Deficit Hyperactivity Disorder (ADHD): Review for Primary Care Clinicians, London Journal of Primary Care. (2010) 3, no. 1, 45–51, 10.1080/17571472.2010.11493296. PMC3960698 25949618

[19] Quintero J. , Gutiérrez-Casares J. R. , and Álamo C. , Molecular Characterisation of the Mechanism of Action of Stimulant Drugs Lisdexamfetamine and Methylphenidate on ADHD Neurobiology: A Review, Neurology and Therapy. (2022) 11, no. 4, 1489–1517, 10.1007/s40120-022-00392-2. 35951288 PMC9588136

[20] Sun C. K. , Tseng P. T. , and Wu C. K. , et al.Therapeutic Effects of Methylphenidate for ADHD in Children With Borderline Intellectual Functioning: A Systematic Review and Meta-Analysis, Scientific Reports. (2019) 9, no. 1, 10.1038/s41598-019-52205-6, 15908. 31685858 PMC6828952

[21] Jaeschke R. R. , Sujkowska E. , and Sowa-Kućma M. , Methylphenidate for ADHD in Adults: A Narrative Review, Psychopharmacology. (2021) 238, no. 10, 2667–2691, 10.1007/s00213-021-05946-0. 34436651 PMC8455398

[22] Malacarne A. , Jain S. , and Barouxis L. , et al.Attention-Deficit Hyperactivity Disorder and Psychostimulant Use in Patients Seeking Dental Care, Journal of Oral Rehabilitation. (2024) 51, no. 6, 947–953, 10.1111/joor.13662. 38379383

[23] Melo G. , Dutra K. L. , and Rodrigues Filho R. , et al.Association Between Psychotropic Medications and Sleep Bruxism: A Systematic Review, Journal of Oral Rehabilitation. (2018) 45, no. 7, 545–554, 10.1111/joor.12633. 29663484

[24] Lavigne G. J. , Khoury S. , Abe S. , Yamaguchi T. , and Raphael K. , Bruxism Physiology and Pathology: An Overview for Clinicians, Journal of Oral Rehabilitation. (2008) 35, no. 7, 476–494, 10.1111/j.1365-2842.2008.01881.x. 18557915

[25] Naguy A. , Elsori D. , and Alamiri B. , Methylphenidate-Induced Nocturnal Bruxism Alleviated by Adjunctive Clonidine, Journal of Child and Adolescent Psychopharmacology. (2019) 29, no. 1, 75–76, 10.1089/cap.2018.0114. 30575406

[26] Tirupathi S. , Afnan L. , and Ruman M. , et al.Can Methylphenidate Use in Children/Adolescents for Treatment of ADHD Lead to Development of Bruxism: Critical Analysis of Literature, Bulletin of Stomatology and Maxillofacial Surgery. (2025) 21, no. 1, 96–103, 10.58240/1829006X-2025.1-96.

[27] Okeson J. P. , Management of Temporomandibular Disorders and Occlusion, 2019, Elsevier.

[28] Francisco A. P. , Lethbridge G. , and Patterson B. , et al.Cannabis Use in ADHD: A Scoping Review, Journal of Psychiatric Research. (2023) 157, 239–256, 10.1016/j.jpsychires.2022.11.029. 36508935

[29] Lobbezoo F. , Ahlberg J. , and Raphael K. G. , et al.International Consensus on the Assessment of Bruxism: Report of a Work in Progress, Journal of Oral Rehabilitation. (2018) 45, no. 11, 837–844, 10.1111/joor.12663. 29926505 PMC6287494

[30] Guimarães L. G. , de Sousa W. V. , and de Andrade Silva S. , et al.Bioactivity and Regenerative Potential of Cannabidiol in Human Dental Pulp Stem Cells: A Scoping Review of in Vitro Studies, The Scientific World Journal. (2026) 2026, no. 1, 10.1155/tswj/6756387, 675638. PMC12937086 41767216

[31] Tsumura M. , Sobhan U. , and Muramatsu T. , et al.TRPV1-Mediated Calcium Signal Couples With Cannabinoid Receptors and Sodium-Calcium Exchangers in Rat Odontoblasts, Cell Calcium. (2012) 52, no. 2, 124–136, 10.1016/j.ceca.2012.05.002. 22656960

[32] Al-Nazhan S. and Al-Shamrani S. , A Radiographic Assessment of the Prevalence of Pulp Stones in Saudi Adults, Saudi Endodontic Journal. (2011) 1, no. 1, 19–26, 10.4103/1658-5984.98312.

[33] González González A. , Martín Casado A. M. , and Gómez Polo C. , Association Between Possible Bruxism, Sleep Quality, Depression, Anxiety and Stress by Gender. A Cross-Sectional Study in a Spanish Sample, Journal of Dentistry. (2025) 156, 10.1016/j.jdent.2025.105677, 105677. 40058482

[34] Zieliński G. , Pająk A. , and Wójcicki M. , Global Prevalence of Sleep Bruxism and Awake Bruxism in Pediatric and Adult Populations: A Systematic Review and Meta-Analysis, Journal of Clinical Medicine. (2024) 13, no. 14, 10.3390/jcm13144259, 4259. 39064299 PMC11278015

